# SPECtre: a spectral coherence-­based classifier of actively translated transcripts from ribosome profiling sequence data

**DOI:** 10.1186/s12859-016-1355-4

**Published:** 2016-11-25

**Authors:** Sang Y. Chun, Caitlin M. Rodriguez, Peter K. Todd, Ryan E. Mills

**Affiliations:** 1 Department of Computational Medicine and Bioinformatics, University of Michigan, Ann Arbor, MI 48109 USA; 2 Department of Neurology, University of Michigan, Ann Arbor, MI 48109 USA; 3 Veterans Affairs Medical Center, Ann Arbor, MI 48105 USA; 4 Department of Human Genetics, University of Michigan, Ann Arbor, MI 48109 USA

**Keywords:** Ribosome profiling, Spectral coherence, Translation, Classification

## Abstract

**Background:**

Active protein translation can be assessed and measured using ribosome profiling sequencing strategies. Prevailing analytical approaches applied to this technology make use of sequence fragment length profiling or reading frame occupancy enrichment to differentiate between active translation and background noise, however they do not consider additional characteristics inherent to the technology which limits their overall accuracy.

**Results:**

Here, we present an analytical tool that models the overall tri­nucleotide periodicity of ribosomal occupancy using a classifier based on spectral coherence. Our software, SPECtre, examines the relationship of normalized ribosome profiling read coverage over a rolling series of windows along a transcript relative to an idealized reference signal without the matched requirement of mRNA-Seq.

**Conclusions:**

A comparison of SPECtre against previously published methods on existing data shows a marked improvement in accuracy for detecting active translation and exhibits overall high accuracy at a low false discovery rate. In addition, SPECtre performs comparably to a recently published method similarly based on spectral coherence, however with reduced runtime and memory requirements. SPECtre is available as an open source software package at https://github.com/mills-lab/spectreok.

**Electronic supplementary material:**

The online version of this article (doi:10.1186/s12859-016-1355-4) contains supplementary material, which is available to authorized users.

## Background

Ribosome profiling is a next-generation sequencing strategy that enriches for ribosome-protected mRNA footprints indicative of active protein translation [6]. Fragments of mRNA bound by ribosomal complexes are selected for by enzymatic digestion, isolated using a sucrose cushion or gradient, released from their occupying ribosome, size-selected by gel electrophoresis, and then sequenced. Thus, sequencing and analysis of ribosome-protected fragments (RPFs) of mRNA enables profiling of the translational content of a sample on a transcriptome-wide level.

Various algorithms have been developed to differentiate protein-coding and non-coding transcripts in ribosome profiling sequence data using fragment length distribution differences [6] and read frame enrichment of aligned reads [2]. However, classification based on extreme outlier analysis of fragment length organization similarity score (FLOSS) differences is agnostic to the ribosome-protected fragment abundance over a transcript. Furthermore, classification based on read frame alignment enrichment (ORFscore) is optimized for canonical open reading frame (ORF) usage only. In addition, neither of the algorithms described above are available as standalone packages and must be implemented by the user. Published more recently, RiboTaper [4] utilizes a coherence-based approach to detect actively translated transcripts from the alignment of ribosome-protected fragments; however, the RiboTaper algorithm requires matched ribosome profiling and mRNA sequence libraries and can take multiple days to analyze a single sample.

**Fig. 1:**
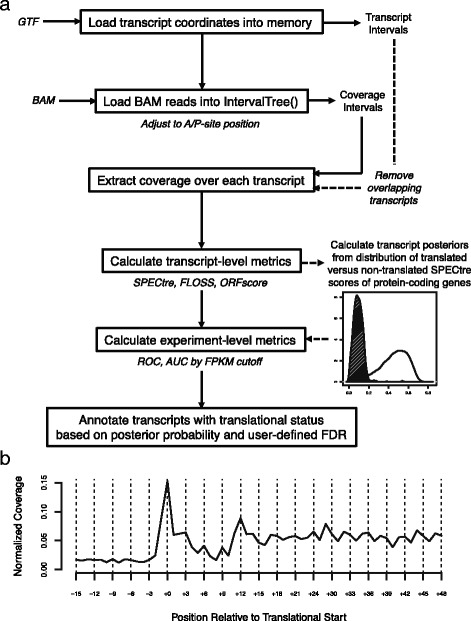
SPECtre pipeline and performance. **a** SPECtre analytical pipeline, input files, formats and outputs. **b** Ribosome profiling read coverage averaged over annotated protein-coding transcripts demonstrates a tri-nucleotide periodic signal characteristic of translation by ribosomes

Here we introduce SPECtre, a classification algorithm based on spectral coherence to identify regions of active translation with high sensitivity and specificity using aligned ribosome profiling sequence reads without the requirement of a matched mRNA sequence library (Fig. 1a). SPECtre leverages a key feature of ribosome profiling where sequence reads aligned to a reference transcriptome will track the tri-nucleotide periodicity characteristic of transcripts as they are translated by ribosomes, and reports both significant signals of translation as well as windowed periodicity scores for visualizing results within a genomic context. Options to change the size of windows analyzed, the step size between adjacent windows, false discovery rate, abundance cutoffs to define actively translated versus non­translated score distributions, and parameters to optimize runtime performance are provided to the user to customize. Implementations of FLOSS and ORFscore are included with SPECtre for comparative purposes.

## Implementation

In contrast to non-coding transcripts, ribosome profiling fragments aligned to protein-coding transcripts are characterized by a tri­nucleotide periodic signal as ribosome-bound mRNAs are translated into protein in a codon-dependent manner (Fig. 1b). Thus coding transcripts may be differentiated from non-coding transcripts by the presence or absence of a strong tri-nucleotide periodic signal. To measure the strength of this tri-nucleotide signal, we calculate the spectral coherence [3] over sliding N nucleotide length windows across a transcript (see also Additional file 1: Materials and Methods). Spectral coherence is a measurement of the power relationship between two signals over the frequency domain, such that two signals with shared frequencies will have high coherence, whereas two unrelated signals will be of low coherence. The SPECtre score, based on a modified Welch’s spectral density estimate [11] of overlapping windows, is calculated for each transcript from a user-provided transcript annotation database.

For a given transcript with coordinates defined by the set *C*, the A- or P-site adjusted read positions overlapping those coordinates are extracted from a BAM alignment file. The coverage over each coordinate in the set is summed, then normalized to the position with the highest coverage, such that all coordinate positions defined by the set *C* range from zero (no coverage) to one (highest coverage). The default SPECtre score is calculated as the average (Welch’s) coherence over *N* nucleotide sliding windows across a normalized coverage region against an idealized tri-nucleotide control signal of the same length. Therefore, the SPECtre score across a normalized coverage region *R,* with coordinates *C*, against an idealized tri-nucleotide periodic signal *S* with frequency *j*, over adjacent *N* nucleotide windows is given by:

1 $$ Spe{c}_{RS,j} = \frac{1}{M}{\displaystyle \sum_{m=1}^M}Co{h}_{R_{m,m+N}{S}_N,j}\ for\  all\ m+N\in C $$

Alternatively, the number of sliding windows (*W*_*n*_) over the coordinate set *C*, may be modified based on the step size between each window. Therefore, given a coordinate set *C*, and step size of *L*:

2 $$ {W}_n={C}_{Ln},\ for\ n\ge 1\  and\ L\ge 1 $$

Therefore, the default SPECtre score of a normalized coverage region *R*, at frequency *j* of an idealized trinucleotide signal *S*, over *N* nucleotide sliding windows with a step size of *L*, is given by the equation:

3 $$ \begin{array}{r}Spe{c}_{RS,j}=\frac{1}{M}{\displaystyle \sum_{m=1}^M}Co{h}_{R_{m,m+N}{S}_N,j\ }\\ {}for\  all\ m\in {W}_n\  and\  all\ m+N\in C\end{array} $$

Distributions of these scores are generated using a user-defined fragments per kilobase per million reads, or FPKM [9], cutoff to differentiate transcripts under active translation from those that are not; these distributions are then used to derive a minimum SPECtre score threshold for active translation given a pre-determined false discovery rate, as well as the posterior probability that a given transcript or region is actively translated.

Ribosome profiling libraries treated with cycloheximide typically isolate RPFs of 28 to 30 nucleotides in length; these fragments align with high fidelity to protein-coding regions [6]. However, in the absence of cycloheximide, conformational changes in the ribosomal complex enrich for a shorter range of RPFs that also map with high fidelity to protein-coding regions [8]. Enrichment of these shorter-range fragments may obscure the tri-nucleotide signal profiled by coherence-based classifiers, like SPECtre, and may under-estimate the number of actively translated ORFs. We simulated increasing variance of RPF lengths outside of the expected enrichment of 28–30 nt length fragments through a biased sampling of reads aligned to the housekeeping gene ACTB. With increased bias, the RPF length distribution is no longer enriched in fragments of 28–30 nt in length, but instead progressively resembles a uniform distribution (Additional file 1: Figure S1). Biased re-sampling of 10,000 out of over 500,000 P-site adjusted reads aligned to ACTB was performed over 10,000 trials, and in each trial the sampled reads were converted into normalized coverage, then scored by SPECtre. Using an extreme outlier cutoff, this biased re-sampling analysis suggests that SPECtre scoring remains robust under increased variance in sequence library fragmentation (Additional file 1: Figure S1 and S2).

## Results

**Fig. 2:**
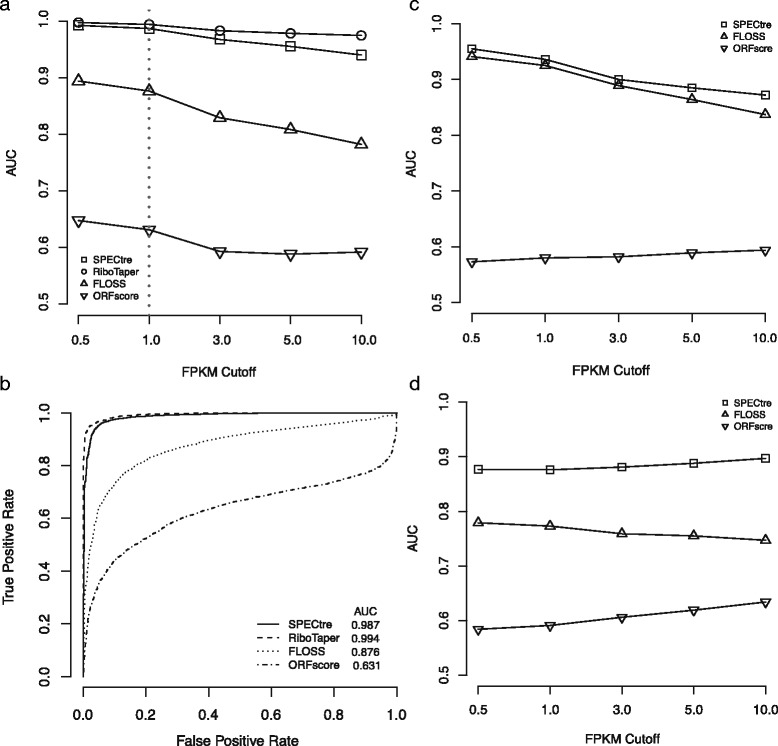
Comparative analysis of SPECtre against previously published translational classification algorithms. **a** Performance of SPECtre, RiboTaper, FLOSS and ORFscore classification of ORF translation at various RPF abundance cutoffs as measured by area under the curve (AUC) in ribosome profiling of HEK293 cells [4]. **b** Receiver operating characteristic (ROC) curves of SPECtre, RiboTaper, FLOSS, and ORFscore at a cutoff of 1.0 FPKM. **c** Performance of SPECtre, FLOSS, and ORFscore classification of ORF translation in ribosome profiling of mESC [7] at various RPF abundance cutoffs as measured by AUC. **d** Performance of SPECtre, FLOSS, and ORFscore classification of ORF translation in a meta-analysis of ribosome profiling in zebrafish [2] over various RPF abundance cutoffs as measured by AUC. All SPECtre analyses were based on 30 nt sliding windows, using a step size of three between each window

We assessed the sensitivity and specificity of each classification algorithm using recently published ribosome profiling and mRNA-Seq data derived from HEK293 cells [4]. For the comparative analysis of each classification algorithm in the HEK293 ribosome profiling library, RiboTaper (version 1.3) was run against published read alignments using the included GENCODE (v19) transcript annotation database [5]. The highest scoring RiboTaper ORFs were extracted from the *orfs_found* results file using the transcript identifiers and scoring method from the *ORFs_max* output. These ORFs were then scored by SPECtre (using default parameters), FLOSS and ORFscore, and then relative performance of each algorithm was assessed by receiver operating characteristic (ROC) analysis. Previous work has benchmarked classifier performance using a series of transcript FPKM cutoffs [4] or other coverage-based metrics [2, 7]. Therefore ROC analyses were performed using a series of ORF abundance cutoffs based on FPKM to differentiate those under active translation from those that are not. In this manner, we are able to assess the ability of each approach to identify ORFs with signatures of active translation in the interrogated cell type. We performed ROC analyses and calculated the area under the curve (AUC) over pre-defined RPF abundance cutoffs (0.5, 1.0, 3.0, 5.0 and 10.0 FPKM) to assess the relative performance of each classification algorithm to accurately define regions of active translation. In HEK293 cells, SPECtre conforms with high fidelity to RiboTaper classification and outperforms both FLOSS and ORFscore to identify actively translated ORFs (Fig. 2a and b).

We also used previously published ribosome profiling data derived from mouse [7] embryonic stem cells (mESC) and zebrafish embryos [2] to assess the performance of SPECtre, FLOSS and ORFscore in the absence of mRNA-Seq data (Additional file 1: Table S1); RiboTaper was excluded from these analyses due to its requirement of matched mRNA-Seq data. Ribosome profiling sequence reads from each set were aligned to the mouse or zebrafish reference genome and transcriptome, respectively. Antisense, overlapping and neighboring protein-coding and non-coding transcripts were removed from the analysis using methods described previously [7]. The FLOSS, ORFscore and SPECtre metrics were calculated for each remaining transcript and ROC analyses were carried out as described above. SPECtre remains robust in its classification of actively translated transcripts in the standalone mESC ribosome profiling library (Fig. 2c and Additional file 1: Table S2), and exhibits a marked improvement in accuracy in a meta-analysis of ribosome profiling libraries derived from zebrafish embryos (Fig. 2d).

**Fig. 3:**
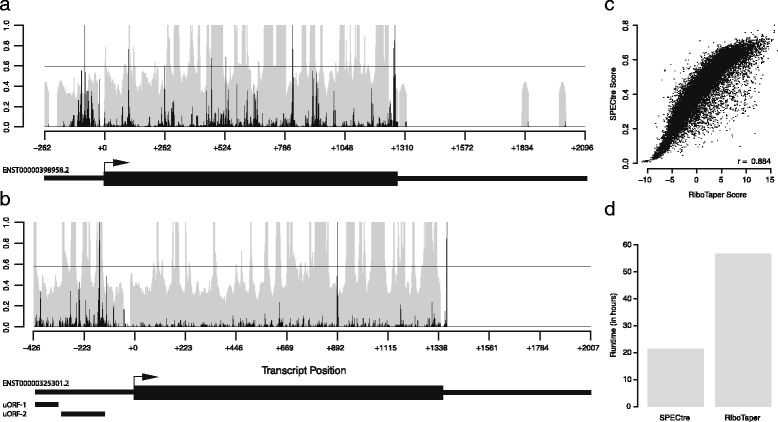
Examples of SPECtre results and runtime comparison to RiboTaper. **a** SPECtre posterior probability profile (*shaded gray*) and normalized P­site adjusted read coverage (*black bars*) over the transcript structure of RCC1­201. Solid, horizontal black line represents the translational threshold as calculated by SPECtre at a false discovery rate of 0.05. Arrow indicates position of annotated translational start site. Thin black boxes (*left to right*) denote the 5′UTR and 3′UTR, respectively, with CDS (*thick black box*) in between. **b** SPECtre posterior probability profile (as above) over the transcript structure of MIEF1. Thin, black boxes under transcript structure denote two uORFs previously identified by RiboTaper analysis. **c** Scatter plot of SPECtre and log2(RiboTaper) scores over assessed ORFs. **d** Comparison of SPECtre (*left*) and RiboTaper (*right*) total compute time, in hours

A unique feature of SPECtre is its ability to report and visualize signals of periodicity in the context of surrounding genomic features. Graphical output from SPECtre analysis is shown for two representative transcripts (Fig. 3a and b). Shown in Fig. 3a is the condensed transcript profile of RCC1­201 (ENST0000398958) with the 5′UTR and 3′UTR depicted by the narrow black lines, and the CDS region depicted with the thicker black line. In gray is the normalized P-site adjusted read coverage over the transcript, with the posterior probability calculated by SPECtre denoted by the black line. The dashed horizontal line represents the translational threshold calculated by SPECtre at a false discovery rate (FDR) of 0.05. In addition to the transcript structure depicted in Fig. 3b are two upstream open reading frames (uORFs) detected separately by RiboTaper (asterisked black bars) in the MIEF1 (ENST0000325301) transcript. Although the 5′UTRs of both RCC1­201 and MIEF1 are profiled by RPF coverage, SPECtre analysis identifies only the uORFs in the 5′UTR of MIEF1, also identified previously by RiboTaper [4], with a tri-nucleotide signal of sufficient strength to be indicative of translational potential.

A further analysis of these and other ORFs assessed by both SPECtre and RiboTaper show a very high degree of score consistency between the two algorithms (Fig. 3c) in addition to their comparable overall accuracy. However, SPECtre has been designed to be fast and efficient and exhibits a runtime almost one-third of that required by RiboTaper (Fig. 3d) without the necessity of RNA-Seq data. This is achieved through SPECtre’s ability to chunk experiments and parallelize analyses over multiple threads, depending on available computational resources, which enables this exceedingly fast runtime relative to existing methods and decreases the computational barrier between library alignment to application and validation. For these experiments, SPECtre analysis was split by chromosome and run using 8 processors, with 32 gigabytes of RAM allocated; RiboTaper was run with default parameters, using 8 processors and 64 gigabyes of RAM. Both SPECtre, and RiboTaper were run on a high-performance computing cluster running Red Hat Enterprise Linux version 6.4 (Santiago). For installation simplicity and application efficiency, SPECtre has been written in Python with minimal third-party dependencies; the only non-standard Python libraries required for SPECtre analysis are RPy2, NumPy [10], HTSeq [1], SAMTools, PyFASTA, PySAM, and the R package ROCR.

## Conclusions

SPECtre is a flexible, lightweight, command-line driven analytical package that identifies regions of active translation through modeling of the tri-nucleotide periodicity characteristic of translation by ribosomes, and does so with high fidelity to a recently published method that relies on a similar coherence-based approach. SPECtre classification also out-performs prevailing algorithms based on fragment length distribution profiling and reading frame occupancy enrichment. SPECtre is robust across ribosome profiling libraries derived from multiple organisms and cell types, even in the absence of matching mRNA-Seq data, and is capable of identifying active translation in regions previously thought to be non-coding. Further, SPECtre is under continuous development to optimize compute runtime and memory overhead in order to facilitate the efficient and accurate investigation of translational dynamics through ribosome profiling sequence analysis.

## Availability and requirements

Project name: SPECtre

Project home page: https://github.com/mills-lab/spectre

Operating systems: Linux, OS X

Programming languages: Python, R

Other requirements: Python v2.7.8+, rpy2, HTSeq, samtools v0.19+, pyfasta v0.5.2+, and pysam 0.9.1.4 + .

## Additional files

Additional file 1: Supplemental methods, tables, figures and example scripts. (DOCX 143 kb)

## Abbreviations

AUC: Area under the curve
FLOSS: Fragment length organization similarity score
FPKM: Fragments per kilobase per million mapped reads
mESC: Mouse embryonic stem cells
ORF: Open reading frame
RAM: Random access memory
RHEL: Red Hat Enterprise Linux
ROC: Receiver operating characteristic
RPF: Ribosome-protected fragment
uORF: Upstream open reading frame
