# Supplementary material for: SPECtre: a spectral coherence-­based classifier of actively translated transcripts from ribosome profiling sequence data
Source: BMC Bioinformatics. 2016 Nov 25;17:482. doi: 10.1186/s12859-016-1355-4 (PMC5123373; doi:10.1186/s12859-016-1355-4)
Supplement: Additional file 1: — Supplemental methods, tables, figures and example scripts. (DOCX 143 kb) [file 12859_2016_1355_MOESM1_ESM.docx]

**Supplementary Online Material**

**SPECtre: a spectral coherence-­based classifier of actively translated transcripts from ribosome profiling sequence data**

Sang Y. Chun, Caitlin M. Rodriguez, Peter K. Todd and Ryan E. Mills

**1 Supplementary Figures**

**Supplemental Table 1.** Number of reads remaining at each stage of pre-processing, alignment and quality filtering of ribosome profiling libraries derived from mESC (Ingolia, 2014) and zebrafish (Bazzini, 2014). The percentages listed are relative to the previous number of reads.

**Supplemental Table 2.** Area under the curve (AUC) values for each classification algorithm at various minimum FPKM cutoffs for ribosome profiling derived from zebrafish (Bazzini, 2014), and mESC (Ingolia, 2014). In addition to the default length of 30 nt sliding windows, 60 nt and 90 nt windows were also tested.

**Supplemental Figure 1.** Read length distribution of all 18 to 40 nt RPFs aligned to the housekeeping gene ACTB in ribosome profiling of mESC (Ingolia, 2014), top left, which depicts the enrichment of 28-30 nt reads indicative of RPFs protected by ribosomes during cycloheximide treatment. Also shown are example read length distribution profiles at various weighted biases (1.0 to 2.0) after sampling 10,000 RPFs from ACTB. As the weighted bias increases from 1.0 to 2.0, the distribution of read lengths increases in variance relative to the experimental read length distribution profile (“All Reads”) and adopts a progressively uniform distribution.

**Supplemental Figure 2.** Distribution of SPECtre scores over the housekeeping gene ACTB after random sampling of 10,000 RPFs with various weighted biases (from 1.0 to 2.0). Dark line inside boxes denotes the median of the SPECtre scores at each weighted bias level, and whiskers depict the minimum and maximum scores. Solid horizontal line denotes the mean SPECtre score over all sampling simulations. The dashed, horizontal lines depict the extreme outlier boundaries as defined by Tukey, which is 1.5 times the inter-quartile range (IQR).

**2 Supplementary Methods**

2.1 Data Retrieval

HEK293 ribosome profiling alignments (Calviello, 2015), in BAM format, were downloaded from the data repository hosted by the authors of RiboTaper (<https://ohlerlab.mdc-berlin.de/files/RiboTaper/alignment_files.tar.gz>). No further pre-processing for the HEK293 alignments was required. Zebrafish ribosome profiling libraries (Bazzini, 2014) were downloaded from the NCBI Gene Expression Omnibus (GEO, accession GSE53693). Ribosome profiling data of mouse embryonic stem cells treated with cycloheximide (Ingolia, 2014) was downloaded from GEO (accession GSE60095, sample GSM1464901).

2.2 Data Pre-processing

Mouse embryonic stem cell and zebrafish ribosome profiling sequence libraries were converted from the Sequence Read Archive (SRA) format to FASTQ. mESC ribosome profiling reads were trimmed of adapters according to previously published methods (Ingolia, 2014). Adapter sequences were removed from zebrafish ribosome profiling reads and further trimmed based on base quality using *fastq-mcf* (Aronesty, 2011). For both trimming methods, a minimum read length of 24 nucleotides was required after adapter removal and supplemental trimming. The minimum threshold for base quality trimming using *fastq-mcf* was set to 10 over a consecutive window of 4 nucleotides. All sequence libraries were then aligned to their respective UCSC (Rosenbloom, 2015) ribosomal RNA contaminant database; mm10 for mESC, and Zv9 for zebrafish embryos. Sequence reads were aligned to their respective ribosomal RNA contaminant database using Bowtie version 1.1.2 (Langmead, 2009) with a seed length of 22 nucleotides, and allowing no mismatches in the seed alignment.

2.3 Alignment

Mouse and zebrafish ribosome profiling reads that did not map to their respective ribosomal RNA contaminant database were aligned using TopHat version 2.0.10 (Kim, 2013). Zebrafish ribosome profiling reads were aligned to the Ensembl (Flicek, 2014) v78 genome and transcriptome references. Mouse embryonic stem cell ribosome profiling sequence reads were aligned to the Ensembl v72 genome and transcriptome reference. All ribosome profiling sequence reads were aligned with TopHat parameters that required Bowtie v1.0.0, Solexa quality scores, no novel junctions to be generated, with a forward/unstranded library type designated.

2.4 Post-alignment Processing

Aligned mESC and zebrafish reads were filtered based on a minimum mapping quality of 10 using SAMtools version 1.2.0 (Li, 2009), and then sorted by genomic position using Picard version 1.114 (<http://broadinstitute.github.io/picard>). For meta-analysis of the zebrafish ribosome profiling data, aligned reads from the sixteen available zebrafish samples and replicates were merged into a single BAM alignment file using Picard. Shell scripts and code specific to each experiment are reproduced in the next section.

**3 SPECtre**

3.1 Read Coverage Normalization

For a given transcript with coordinates defined by the set *C*, the A- or P-site adjusted read positions overlapping those coordinates are extracted from a BAM alignment file. The coverage over each coordinate position in the set is summed, then normalized to the highest coverage such that all coordinate positions defined by set *C* range from zero (no coverage) to one (highest coverage).

3.2 Spectral Coherence

In signal processing, coherence measures the power relationship between two signals as a function of frequency. Coherence estimates range from zero, where two signals are fully independent of each other, to one, where one signal may be perfectly predicted by the other. Assuming a sampling interval **Δ** over time interval *T*, signal *X_j_* and its Fast Fourier Transform (FFT) *X_j_^*^* define the power spectrum of signal *X* at frequency *j* as:

*(1)*

$$S_{XX,j}= \left( \frac{{2\Delta}^{2}}{T} \right)X_{j}X_{j}^{*}$$

The cross-power spectrum of signal *X_j_* and Y_j_ at frequency *j,* is calculated as the mean of the product of signal *X_j_* and the Fast Fourier Transform of signal _j_ over *K* trials:

*(2)*

$$<S_{XY,j}> = \left( \frac{{2\Delta}^{2}}{T} \right)\left( \frac{1}{K} \right)\sum_{k=1}^{K} X_{j,k}Y_{j,k}^{*}$$

Coherence is defined as the magnitude of the cross-power spectrum between signal *X* and *Y* at frequency *j* divided by the product of the square roots of the power spectrum of signal *X* at frequency *j*, and the power spectrum of signal *Y* at frequency *j*.

*(3)*

$${Coh}_{XY, j}= \frac{\left| <S_{XY,j}> \right|}{\sqrt{<S_{XX,j}>}\sqrt{<S_{YY,j}>}}$$

3.3 SPECtre Score

The default SPECtre score is calculated as the average (Welch, 1967) coherence over *N* nucleotide sliding windows across a normalized coverage region against an idealized tri-nucleotide control signal of the same length. Welch’s coherence decreases the variance of the coherence estimate at the expense of resolution. Alternatively, modified Welch’s coherence estimates over a region may be calculated using the median, maximum, or the non-zero mean or median. The SPECtre score of a normalized coverage region *R* with coordinates *C*, at frequency *j* against an idealized tri-nucleotide signal *S*, over adjacent *N* nucleotide windows is given by:

*(4)*

$${Spec}_{RS,j}= \frac{1}{M}\sum_{m=1}^{M} {Coh}_{R_{m,m+N}S_{N},j} for all m+N\in C$$

The number of sliding windows over the coordinate set *C* may be modified based on the step size between each window. Given a coordinate set *C*, and step size of *L:*

*(5)*

$$W_{n}=C_{Ln}, for n\geq1 and L\geq1$$

Therefore, the default SPECtre score of a normalized coverage region *R*, at frequency *j* against an idealized tri-nucleotide signal *S*, over *N* nucleotide windows with a step size of *L* is:

*(6)*

$${Spec}_{RS,j}= \frac{1}{M}\sum_{m=1}^{M} {Coh}_{R_{m,m+N}S_{N},j} for all m\in W_{n} and all m+N\in C$$

3.4 Read Length Bias

Treatment with cycloheximide typically isolates ribosome-protected fragments 28 to 30 nucleotides in length, which align with high fidelity to regions annotated to protein-coding transcripts (Ingolia, 2009). However, in the absence of cycloheximide, conformational changes in the ribosomal complex may enrich for a shorter range of RPFs that also map with high-fidelity to regions annotated to protein-coding transcripts (Lareau, 2014). It is possible that these shorter length RPFs may obscure the tri-nucleotide signal of longer length RPFs that may cause coherence-based classifiers, like SPECtre, to under-estimate the number of actively translated ORFs in a ribosome profiling experiment. Ideally, this could be tested using simulated data as is done for whole genome sequencing using *wgsim* (<https://github.com/lh3/wgsim>) or RNA-Seq (Frazee, 2015). However, unlike RNA-Seq, simulation of RPFs would have to account for the distribution of RPFs protected by ribosomes (Supplemental Figure 1, see “All Reads”) as well as variance in the tri-nucleotide periodicity signal once those RPFs are aligned to the transcriptome. Instead of simulating an entire ribosome profiling experiment, we have examined the robustness of SPECtre scoring as a function of increased variance in RPF lengths outside of the expected 28-30 nt range. We have simulated this by randomly sampling 10,000 RPFs from the over 500,000 mESC RPFs (Ingolia, 2014) aligned to the housekeeping gene ACTB using a weighted biased probability function.

Given a distribution of aligned RPF lengths*, D*, in a ribosome profiling experiment, with the RPF lengths defined by the set, *L = {18, 19, 20, …, 38, 39, 40}*, and the relative frequency of each RPF length given by *p_Ln_,* we define the weighted bias for a given RPF length to be randomly sampled as:

*(7)*

$$W_{Ln}=\frac{p_{Ln}}{p_{Ln}^{b}}$$

Where *b* is the bias assigned to the sampling distribution, such that if *b = 1* the weighted bias for a given RPF length to be randomly sampled would be defined by the experimental RPF length frequencies. In contrast, if *b = 2*, the weighted bias for a given RPF length to be randomly sampled would be defined by the inverse of the experimental RPF length frequencies. The effect of increasing *b* from 1.0 to 2.0 may be seen in Supplemental Figure 1; starting with *b = 1*, the random sampling (with replacement) of 10,000 reads from the ~500,000 RPFs aligned to ACTB closely conforms to the experimental RPF length distribution (Supplemental Figure 1, see “All Reads”). As *b* is increased from 1.0 to 2.0, the RPF length distribution demonstrates increased variance in RPF lengths outside of the expected enrichment of 28-30 nt fragments to the extent that the RPF length distribution progressively resembles a uniform distribution.

Incrementing *b* from 1.0 to 2.0, we sampled 10,000 RPFs from ~500,000 aligned to ACTB with replacement using the *sample()* function in R. Sampling was performed with replacement due to the low number of RPFs at the low and high extremes, and to simulate the persistence of sequence duplication. This biased re-sampling was done over 10,000 trials, and in each trial the normalized read coverage over ACTB was calculated then scored using SPECtre against an idealized tri-nucleotide periodic signal of the same length. The results of these biased sampling simulations are shown in Supplemental Figure 2; as *b* is increased from 1.0 to 2.0, the distribution of SPECtre scores is plotted with the median score denoted by the dark black inside each box, and the extremities depicted by the ends of each whisker. The horizontal black line represents the mean SPECtre score over all simulations; the dashed lines above and below mark the boundaries of the extreme outlier cutoff as defined by Tukey. Tukey’s outlier cutoffs are defined as 1.5 times the inter-quartile range. Base on this outlier analysis of 10,000 trials over an increasing weighted bias for RPF length selection, SPECtre is robust against increasing variance in RPF lengths outside of the expected (28-30 nt) range.

**4 Experimental Scripts**

4.1 Adapter Removal and Trimming

# For mESC libraries:

fastx_clipper -Q33 -a CTGTAGGCACCATCAAT -l 24 -c -n –v –i /path/to/FASTQ > clipped.fq

fastx_trimmer -Q33 -f 2 -m 24 -i /path/to/clipped.fq > trimmed.fq

# For zebrafish ribosome profiling library:

fastq-mcf -o /path/to/trimmed.fq -l 24 -q 10 -w 4 -t 0 /path/to/adapter.fa /path/to/FASTQ

4.2 Alignment to rRNA Contaminant Database

bowtie -l 22 -n 0 -S --un ribo-rRNA.fq /path/to/rRNA_index /path/to/trimmed.fq > rRNA.sam

4.3 Alignment to Reference Transcript and Genome

tophat --bowtie1 --solexa-quals --no-novel-juncs --library-type fr-unstranded \

--GTF /path/to/GTF –o /path/to/alignments /path/to/genome_index /path/to/ribo-rRNA.fq

4.4 Alignment Post-processing

samtools view –b –q 10 /path/to/alignments/accepted_hits.bam > filtered_hits.bam

samtools index filtered_hits.bam

**5 Example Analysis**

5.1 Test Data

The test data available on the SPECtre GitHub repository consists of human SH-SY5Y neuroblastoma ribosome profiling (treated with cycloheximide) sequence alignments limited to the Ensembl v78 human chromosome 3 genome and transcriptome references. Likewise, the annotation database is limited to human chromosome 3 for testing purposes.

5.2 Test Analysis

python /path/to/SPECTre.py \

--input /path/to/test.bam \

--output /path/to/spectre_test.txt \

--log /path/to/spectre_test.log \

--gtf /path/to/Homo_sapiens.GRCh38.78.test.gtf \

--fpkm /path/to/isoforms.fpkm_tracking \

--len 30 \

--fdr 0.05 \

--min 3.0 \

--type mean \

--floss \

--orfscore

5.3 Cluster Script

For faster runtime, SPECtre may be parallelized and submitted to a compute cluster. A sample PBS script is provided below:

#!/bin/bash

#PBS -N spectre_test

#PBS -l nodes=8,mem=32gb,walltime=96:00:00

#PBS -m abe

#PBS -M stonyc\@umich.edu

#PBS -d .

#PBS -V

#PBS -o spectre_test.out

#PBS -e spectre_test.err

python /path/to/SPECTre.py \

--input /path/to/test.bam \

--output /path/to/spectre_test.txt \

--log /path/to/spectre_test.log \

--gtf /path/to/Homo_sapiens.GRCh38.78.test.gtf \

--fpkm /path/to/isoforms.fpkm_tracking \

--nt 8 \ # use up to 8 processors

--len 30 \

--fdr 0.05 \

--min 3.0 \

--type mean \

--floss \

--orfscore

5.4 Weighted Bias Sampling

For increasing bias*, b*, RPFs aligned to ACTB are sampled, converted into a normalized coverage vector, and then scored using SPECtre. The R script for a single bias is shown below, which can be run in parallel with other biases for faster runtime and efficiency:

# LOAD READS INTO R:

reads <- read.delim("/dir/to/ACTB_reads.txt", stringsAsFactors=FALSE)

read_coverage <- function(positions.vector) {

coverage <- rep(0, times=1128)

coverage.table <- table(positions.vector)

positions = as.numeric(names(coverage.table))

depth = as.vector(coverage.table)

for (i in 1:length(positions)) {

coverage[positions[i]] <- depth[i]

}

return(coverage)

}

normalized_coverage <- function(cov) {

return(cov/max(cov))

}

roundup <- function(x, to=3) {

to*(x%/%to + as.logical(x%%to))

}

calculate_spectre_score <- function(normalized.coverage, window.size, step.size) {

coherences <- numeric()

coding.coverage <- rep(c(4/6,1/6,1/6), times=roundup(length(normalized.coverage)/3))[1:length(normalized.coverage)]

for (i in seq(1, length(normalized.coverage)-30, 3)) {

j = i + window.size

if (sum(normalized.coverage[i:j]) == 0 || is.na(sum(normalized.coverage[i:j]))) {

coherences <- c(coherences, 0.0)

} else {

test.spec <- spec.pgram(data.frame(normalized.coverage[i:j], coding.coverage[i:j]), spans=c(3,3), plot=FALSE)

coherences <- c(coherences, test.spec$coh[which(abs(test.spec$freq-1/3) == min(abs(test.spec$freq-1/3)))])

}

}

return(mean(coherences))

}

sample_reads <- function(df, sample.size, weight) {

reads <- data.frame(name=rep(NA, times=sample.size), len=rep(NA, times=sample.size), pos=rep(NA, times=sample.size))

names <- sample(df$read, size=sample.size, replace=TRUE, prob=df$p/(df$p^weight))

for (i in 1:length(names)) {

read.name <- unlist(strsplit(names[i], split="\\|"))[1]

read.len <- as.numeric(unlist(strsplit(names[i], split="\\|"))[2])

read.pos <- as.numeric(unlist(strsplit(names[i], split="\\|"))[3])

reads[i,"name"] <- read.name

reads[i,"len"] <- read.len

reads[i,"pos"] <- read.pos

}

return(reads)

}

reads.dist <- data.frame(c(18:40))

colnames(reads.dist) <- c("len")

# CALCULATE DISTRIBUTION OF READ LENGTHS:

reads.dist$num <- NA

reads.dist$ratio <- NA

for (i in 1:length(reads.dist[,1])) {

reads.dist[i,"num"] <- length(reads$len[reads$len==reads.dist[i,"len"]])

reads.dist[i,"ratio"] <- length(reads$len[reads$len==reads.dist[i,"len"]])/length(reads$len)

}

# INITIALIZE THE SCORING MATRIX:

scores <- numeric()

weight <- 1.0

# CALCULATE SPECTRE SCORE FOR VARIABLY BIASED SAMPLING OF READ LENGTHS OVER ACTB:

n.trials = 10000 # Number of trials.

n.sample = 10000 # Sample size.

for (i in 1:n.trials) {

sampled.reads <- sample_reads(reads, n.sample, weight)

sampled.coverage <- read_coverage(sampled.reads$pos)

sampled.coverage.nlz <- normalized_coverage(sampled.coverage)

sampled.spec <- calculate_spectre_score(sampled.coverage.nlz, 30, 3)

scores[i] <- sampled.spec

print(paste(Sys.time(), paste(weight, i, sep=": "), sep=" "))

}

**6 Data**

mESC (Ingolia, 2014), GSE53693

Zebrafish (Bazzini, 2014), GSE60095, sample GSM1464901

HEK293 (Calviello, 2015), <https://ohlerlab.mdc-berlin.de/files/RiboTaper/alignment_files.tar.gz>

**7 Usage and Implementation**

7.1 Usage

The files required for SPECtre analysis are an indexed alignment file in BAM format, an isoform-level expression tracking file output from Cufflinks (Trapnell, 2010), and a transcript annotation file in the form of a Gene Transfer Format (GTF, version 2.2+) file. GTF annotation files may be downloaded from the UCSC Genome Browser (Rosenbloom, 2010) or the Ensembl archive (Flicek, 2014). User-defined arguments to specify the SPECtre scoring method (mean, median, maximum, etc.), the length of the windows over which to calculate the spectral coherence, minimum FPKM cutoffs, and FDR thresholds to calculate the Bayesian posterior probability of translation for each transcript are provided. Implementations of the FLOSS metric and ORFscore have also been made available as optional command-line arguments. Finally, an option to calculate the un-windowed spectral coherence of the full length of a transcript has been provided.

7.2 Output

Depending on the detail requested, the end-user will be provided with a tab-delimited text document with annotation information relevant to each transcript tested, including a unique identifier, genomic coordinates of the CDS and UTR regions, transcript abundance, the normalized read coverage over each region, the user-defined spectral coherence metric, and the Bayesian posterior probability of each transcript to be classified as actively translated. Optionally, the respective fragment length distribution and FLOSS metric, and the total number of reads over each frame and ORFscore may be calculated and output for each transcript. The spectral coherence score distribution for translated versus non-translated transcripts, and summary ROC and AUC plots are generated for user review. All plots generated by the SPECtre analytical package are output in PDF format.

7.3 Implementation

SPECtre is a standalone analytical package written in Python, and is intended to run on a wide range of platforms. Therefore, installation of only a limited number of non-standard modules is required. SPECtre requires the following non-standard modules: NumPy (van der Walt, 2011) and RPy2 (<http://rpy.sourceforge.net>), and HTSeq (Anders, 2015). HTSeq is used to convert alignments from the BAM or SAM input into transcript coverage, hash transcript intervals, and check for overlaps. Prior installation of R and the ROCR package (Sing, 2005) are required to perform the ROC analyses and generate summary plots. Shell scripts and SPECtre analysis for typical single sample and multi-sample comparative analyses are available as Supplementary Material and via the SPECtre GitHub repository.

**References**

Aronesty, E. ea-utils: Command-line tools for processing biological sequencing data. 2011. <http://code.google.com/p/ea-utils>

Bazzini AA, Johnstone TG, Christiano R, Mackowiak SD, Obermayer B, Fleming ES, Vejnar CE, Lee MT, Rajewsky N, Walther TC, Giraldez AJ. Identification of small ORFs in vertebrates using ribosome footprinting and evolutionary conservation. *EMBO J*. 2014; 33, 981-993.

Calviello L, Mukherjee N, Wyler E, Zauber H, Hirsekorn A, Selbach M, Landthaler M, Obermayer B, Ohler U. Detecting actively translated open reading frames in ribosome profiling data. *Nat Methods*. 2015; 13, 165-170.

Flicek P, Amode MR, Barrell D, Beal K, Billis K, Brent S, Carvalho-Silva D, Clapham P, Coates G, Fitzgerald S, Gil L, Girón CG, Gordon L, Hourlier T, Hunt S, Johnson N, Juettemann T, Kähäri AK, Keenan S, Kulesha E, Martin FJ, Maurel T, McLaren WM, Murphy DN, Nag R, Overduin B, Pignatelli M, Pritchard B, Pritchard E, Riat HS, Ruffier M, Sheppard D, Taylor K, Thormann A, Trevanion SJ, Vullo A, Wilder SP, Wilson M, Zadissa A, Aken BL, Birney E, Cunningham F, Harrow J, Herrero J, Hubbard TJ, Kinsella R, Muffato M, Parker A, Spudich G, Yates A, Zerbino DR, Searle SM. Ensembl 2014. *Nucleic Acids Res*. 2014; 42, 749-55.

Frazee AC, Jaffe AE, Langmed B, Leek JT. Polyester: simulating RNA-seq datasets with differential transcript expression. *Bioinformatics*. 2015; 31: 17*,* 2778-84.

Ingolia NT, Ghaemmaghami S, Newman JRS, Weissman JS. Genome­wide analysis in vivo of translation with nucleotide resolution using ribosome profiling. *Science*. 2009; 324, 218­223.

Ingolia NT, Brar GA, Stern-Ginossar N, Harris MS, Talhouame GJS, Jackson SE, Wills MR, Weissman JS. Ribosome profiling reveals pervasive translation outside of annotated protein-coding genes. *Cell Reports*. 2014*;* 8, 1365-1379.

Kim D, Pertea G, Trapnell C, Pimentel H, Kelley R, Salzberg SL. TopHat2: Accurate aligment of transcriptomes in the presence of insertions, deletions and gene fusions. *Genome Biol*. 2013; 14: R36.

Langmead B, Trapnell C, Pop M, Salzberg SL. Ultrafast and memory-efficient alignment of short DNA sequences to the human genome. *Genome Biol*. 2009; 10(3): R25.

Lareau LF, Hite DH, Hogan GJ, Brown PO. Distinct stages of the translation elongation cycle revealed by sequencing ribosome-protected mRNA fragments. *eLife*. 2014; 3: e01257.

Li H, Handsaker B, Wysoker A, Fennell T, Ruan J, Homer N, Marth G, Abecasis G, Durbin R. The sequence alignment/map (SAM) format and SAMtools. *Bioinformatics*. 2009; 25, 2078-9.

Rosenbloom KR, Armstrong J, Barber GP, Casper J, Clawson H, Diekhans M, Dreszer TR, Fujita PA, Guruvadoo L, Haeussler M, Harte RA, Heitner S, Hickey G, Hinrichs AS, Hubley R, Karolchik D, Learned K, Lee BT, Li CH, Miga KH, Nguyen N, Paten B, Raney BJ, Smit AF, Speir ML, Zweig AS, Haussler D, Kuhn RM, Kent WJ. The UCSC Genome Browser Database: 2015 update. *Nucleic Acids Res*. 2015; 43, 670-81.

Sing T, Sander O, Beerenwinkel N, Lengauer T. ROCR: Visualizing classifier performance in R. *Bioinformatics*. 2005; 21, 3940-3941.

Welch P. The use of fast Fourier transform for the estimation of power spectra: A method based on time averaging over short, modified periodograms. *IEEE Transactions on Audio and Electroacoustics*. 1967; 15(2), 70-73.
